# Survival trends and prognostic factors of patients with newly diagnosed multiple myeloma accompanied with extramedullary disease

**DOI:** 10.1080/07853890.2023.2281657

**Published:** 2023-12-12

**Authors:** Jing Wang, Na Shen, Xuxing Shen, Run Zhang, Yuanyuan Jin, Jianyong Li, Lijuan Chen

**Affiliations:** Department of Hematology, Jiangsu Province Hospital, The First Affiliated Hospital of Nanjing Medical University, Nanjing, China

**Keywords:** Multiple myeloma, extramedullary disease, clinical characteristics, prognostic model

## Abstract

**Background:** Extramedullary disease (EMD) is an unusual event in patients with MM. This study aimed to assess the prognostic impact of EMD and develop an EMD-based risk model to estimate the survival of patients with newly diagnosed multiple myeloma (NDMM).

**Methods: **A total of 518 patients were enrolled in this study, of which 121 presented with EMD at the initial diagnosis. Patients were divided into non-EMD, extramedullary-bone-related (EM-B) and extramedullary-extraosseous (EM-E) groups. Clinical characteristics were compared using the chi-squared test or Fisher’s exact test. Survival curves were plotted using the Kaplan-Meier method, and a nomogram was constructed based on the Cox proportional hazards model.

**Results: **Compared to patients without EMDs, patients with EM-E were younger (*p* = 0.028), and those with EM-B had less renal damage (*p* < 0.001). The EM-E group had the worst progression-free survival (PFS) and overall survival (OS). In addition, patients with multiple sites of EMD invasion or high Ki67 expression had poor OS. Lenalidomide-based treatment showed the worst outcome, and autologous stem cell transplantation (ASCT) remarkably improved the survival of patients with EMD. A prognostic model (MM prognostic index, MM-PI) comprising lactate dehydrogenase (LDH), circulating plasma cells (CPC), del(17p), and type of extramedullary involvement was developed, and a 4-factor nomogram.

**Conclusions:** We established a risk model incorporating extramedullary disease that provides accurate and individualized survival estimates for patients with NDMM.

## Introduction

Plasma cell disease is a group of diseases characterized by abnormal proliferation of monoclonal plasma cells from mature B lymphocytes, accompanied by the secretion of excessive monoclonal immunoglobulin or polypeptide light/heavy chains, including multiple myeloma (MM), extramedullary lesions (EMDs), and plasma cell leukemia (PCL) [1, 2]. MM is characterized by neoplastic plasma cells localized in the bone marrow [3]. However, a small percentage of patients present with clonal plasma cells separating from the bone marrow and then infiltrating the soft tissues adjacent to or away from the bone at the time of first diagnosis, progression, or recurrence, termed as EMDs. Based on the site of the lesions, EMD is classified into two types: extramedullary-bone-related (EM-B) and extramedullary-extraosseous (EM-E). EM-B refers to plasmacytomas that extend *via* the bone cortex from a site of localized bone destruction and tumor masses formed by cells disseminating hematogenously to the skin, liver, lymph nodes and central nervous systems, respectively [2, 4, 5].

The incidence of EMD is 7% at initial diagnosis and 6–20% at recurrence [6–9]. 85% of these patients have EM-B, and the median age of patients with EM-E is lower than that of patients with EM-B [9, 10]. Patients with more aggressive phenotypes, including high lactate dehydrogenase (LDH) levels, osteolytic lesions, and hypercalcemia, have a higher risk of developing EMD [7, 11–13]. Other high-risk cytogenetics involving del(17p), t(4;14) and t(14;16) are risk factors for the development [14]. The incidence of EMD is elevated, although the survival rate of MM is prolonged [7]. In the era of novel drugs and regimens, treatments such as carfilzomib, pomalidomide, autologous stem cell transplantation (ASCT), and chimeric antigen receptor T-cell (CAR-T) immunotherapy have finite roles in EMDs [11, 15–18]. Even the limited efficacy of daratumumab has been identified in patients with EMD [19].

In the current study, we analyzed the baseline characteristics of patients with EMD, which could partly reflect the features of these populations. We also evaluated the prognostic variables in patients with EMD. Based on these prognostic factors, we established a prognostic model to predict the survival of MM patients with EMDs.

## Methods

### Patient population

A total of 518 patients consecutively diagnosed with MM from January 2013 to December 2021 at the First Affiliated Hospital of Nanjing Medical University were enrolled in the study. Patients diagnosed with solitary extramedullary plasmacytoma, monoclonal gammopathy of undetermined significance (MGUS), smoldering MM, and plasma cell leukemia were excluded from this study. Data including ISS stage, gender, age, M protein type and the level of bone marrow plasma cell (BMPC), corrected serum calcium (Ca), β2-microglobulin (β2-MG), lactate dehydrogenase (LDH), serum creatinine (Cr), albumin (ALB), and hemoglobin (HB) were available for all patients. Circulating plasma cell (CPC) quantified by flow cytometry in the peripheral blood was available for 416 patients, and the cut-off value of CPC was based on our previous study [20]. Cytogenetics at diagnosis were available for 362 patients in whom the del(17p), 1q21 gain and IgH translocations, including t(4;14), t(14;16) and t(11;14) were detected. EMD is typically diagnosed by computed tomography (CT), positron emission tomography (PET)/CT, magnetic resonance imaging (MRI), or other imaging examinations. Definitions of diagnosis, categorical responses, and myeloma relapse were based on the International Myeloma Working Group criteria [3, 21]. This study was approved by the Ethics Committee of the First Affiliated Hospital of Nanjing Medical University (No. 2022-SR-448). Informed consents were obtained from all patients before enrollment into the study.

### Survival analysis

The median follow-up period was 41.0 months until 30 June 2022. Overall survival (OS) was defined as the time from diagnosis to death or last follow-up. Progression-free survival (PFS) was defined as the time from diagnosis to the first progression, relapse, or final follow-up.

### Establishment of a predictive nomogram

LDH, del(17p) and CPC at diagnosis were available for 316 patients. A nomogram including type of extramedullary, LDH, del(17p) and CPC was developed based on those 316 patients using Cox’s proportional hazards models. The calibration curves reflect the consistency between the actual outcomes and nomogram-predicted probabilities. Internal validation of the model was performed using a bootstrap approach with 1000 replications.

### Statistical methodology

Categorical variables were compared among the three groups using Fisher’s exact test or chi-square test. The Kaplan-Meier algorithm was used to plot survival curves. The log-rank test was used to assess variation in survival. The *p*-value was 2-sided and *p* < 0.05 was considered to be statistically significant. All analyses were conducted using SPSS Version 26.0, GraphPad Prism 9.0, and R software (version 3.6.1).

## Results

### Involvements of EMD

A total of 518 patients were enrolled in this study, of which 121 (23.4%) presented with EMD at the initial diagnosis. Among all EMD cases, 22 were defined as EM-E, 91 as EM-B and 8 as both EM-E and EM-B. Patients who presented with both EM-E and EM-B were categorized into EM-E group. The most common sites of EM-B are the vertebrae, ribs, pelvis, sternum and skull, whereas the skin, subcutaneous soft tissue, and pleural involvement are more predominant in EM-E. More than half of the patients (76/121) with EMD had invasion at one site, while 25.6% (31/121) of patients had invasion at more than three sites. The anatomical distributions of EMD are shown in Table 1 in detail.

**Table 1.** Sites of extramedullary disease.

Anatomic site	EM-B (*n* = 99)	Anatomic site	EM-E (*n* = 30)
Vertebra	46	Skin and subcutaneous soft tissue	12
Rib	42	Pleura	10
Pelvis	24	Lung	3
Skull	11	Lymph node	2
Sternum	11	Mediastinum	2
Humerus	8	Larynx	1
Femur	7	Liver	1
Scapula	6	Meninges	1
Clavicle	3	Pituitary	1
Tibia	2	Paranasal sinus	1
Fibula	1		

### Baseline clinical features of MM patients with EMD

We divided all patients into three subgroups: non-EMD, EM-B and EM-E. Patients with EM-B or EM-E had significantly lower ALB and HB levels than those in the non-EMD group. Compared to patients without EMDs, patients with EM-E were younger (*p* = 0.028), and patients with EM-B had less renal damage (*p* < 0.001). Cytogenetics at diagnosis was available for 362 patients. However, no significant differences were found between the three subgroups, although t(4;14) showed a slight association with EM-B occurrence. Ki-67 expression in the extramedullary lesions was measured using immunohistochemistry. According to the cut-off criteria calculated using the ROC curve, 52 patients with higher levels of Ki-67 expression (>15%) and 28 patients with lower levels (≤15%) were determined. The percentage of patients with high Ki-67 scores was higher in the EM-E group (18/22, 82%) than in the EM-B group (34/58, 59%) (Table 2).

**Table 2.** Baseline characteristics of patients.

	non-EMD (*n* = 397)	EM-B (*n* = 91)	EM-E (*n* = 30)	P1	P2	P3
Age				0.196	**0.028**	0.178
≤65 years	237 (59.7%)	61 (67.0%)	24 (80.0%)			
>65 years	160 (40.3%)	30 (33.0%)	6 (20.0%)			
Gender				0.174	0.937	0.489
Male	222 (55.9%)	33 (36.3%)	17 (56.7%)			
Female	175 (44.1%)	58 (63.7%)	13 (43.3%)			
Type of M protein				**0.006**	0.079	0.438
IgG	203(51.1%)	44(48.4%)	11(36.7%)			
IgA	98(24.7%)	11(12.1%)	6(20.0%)			
Light chain	77(19.4%)	29(31.9)	9(30.0%)			
Others	19(4.8%)	7(7.7%)	4(13.3%)			
ISS stage				**<0.001**	0.184	0.672
I	49 (12.3%)	29 (31.9%)	7 (23.3%)			
II	144 (36.3%)	29 (31.9%)	11 (36.7%)			
III	204 (51.4%)	33 (36.3%)	12 (40.0%)			
R-ISS stage				0.182	0.191	0.757
I	28 (8.1%)	11 (14.5%)	4 (15.4%)			
II	256 (73.8%)	50 (65.8%)	15 (57.7%)			
III	63 (18.1%)	15 (19.7%)	7 (26.9%)			
LDH (IU/L)				0.570	0.144	0.382
Normal	353 (88.9%)	79 (86.8%)	24 (80.0%)			
Elevated	44 (11.1%)	12 (13.2%)	6 (20.0%)			
Cr(umol/L)				**<0.001**	0.085	0.408
≤177	303 (76.3%)	86 (94.5%)	27 (90.0%)			
>177	94 (23.7%)	5 (5.5%)	3 (10.0%)			
Ca(mmol/L)				0.360	1.000	0.780
≤2.75	346 (87.2%)	76 (83.5%)	26 (86.7%)			
>2.75	51 (12.8%)	15 (16.5%)	4 (13.3%)			
BMPC (%)				0.194	0.593	0.305
≤60	342 (86.1%)	83 (91.2%)	25 (83.3%)			
>60	55 (13.9%)	8 (8.8%)	5 (16.7%)			
ALB (g/L)				**<0.001**	**0.041**	0.563
<35	259 (65.2%)	37 (40.7%)	14 (46.7%)			
≥35	138 (34.8%)	54 (59.3%)	16 (53.3%)			
HB (g/L)				**<0.001**	**0.003**	0.796
<100	277 (69.8%)	37 (40.7%)	13 (43.3%)			
≥100	120 (30.2%)	54 (59.3%)	17 (56.7%)			
CPC (%)				0.096	0.080	0.548
<0.105	212 (66.0%)	57 (76.0%)	17 (85.0%)			
≥0.105	109 (34.0%)	18 (24.0%)	3 (15.0%)			
Cytogenetics						
1q21 gain	141 (50.7%)	30 (47.6%)	14 (66.7%)	0.657	0.158	0.130
Del(17p)	27 (9.7%)	7 (11.1%)	4 (19.0%)	0.738	0.252	0.455
t(4;14)	45 (16.2%)	16 (25.4%)	1 (4.8%)	0.085	0.218	0.058
t(14;16)	8 (2.9%)	0	1 (4.8%)	0.360	1.000	0.250
t(11;14)	25 (9%)	8 (12.7%)	2 (9.5%)	0.369	1.000	1.000
ASCT				0.091	0.539	0.161
Yes	85 (21.4%)	27 (29.7%)	5 (16.7%)			
No	312 (78.6%)	64 (70.3%)	25 (83.3%)			

P_1_, non-EMD vs. EM-B; P_2_, non-EMD vs. EM-E; P_3_, EM-B vs. EM-E; ISS, International Staging System; R-ISS, Revised International Staging System; LDH, lactate dehydrogenase; Cr, creatinine; Ca, corrected serum calcium; ALB, albumin; HB, hemoglobin; BMPC, bone marrow plasma cells; CPC, circulating plasma cells; ASCT, autologous stem cell transplantation. Statistically significant *p*-values are written in bold.

The best response of patients with EMD was available for 116 patients, of which 29 achieved stringent CR (sCR), 28 achieved complete remission (CR), 22 achieved very good partial remission (VGPR) and 21 achieved partial remission (PR). As for response, 89.8% (79/88) of patients in the EM-B group and 75.0% (21/28) of patients in the EM-E group achieved PR or better (*p* = 0.062) (Table 3).

**Table 3.** Response of patients with EMD.

Response	EM-B (*n* = 88)	EM-E (*n* = 28)	*p* value
sCR (%)	25 (28.4%)	4 (14.3%)	
CR (%)	21 (23.9%)	7 (25.0%)	
VGPR (%)	19 (21.6%)	3 (10.7%)	
PR (%)	14 (15.9%)	7 (25.0%)	
ORR (%)	79 (89.8%)	21 (75.0%)	0.062

sCR, stringent complete remission; CR, complete remission; VGPR, very good partialremission remission; PR: partial remission; ORR: overall response rate.

### Survival trends of patients with non-EMD, EM-B and EM-E

**Figure 1.**
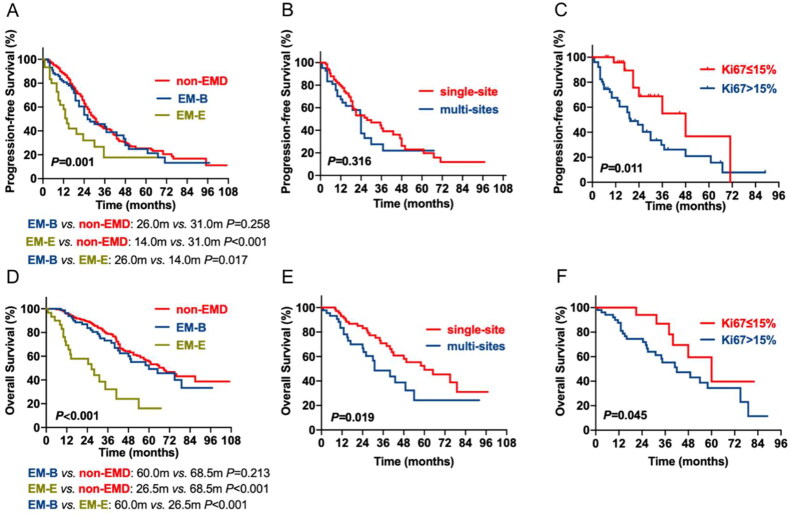
The impact of EMD, the number of invasion sites and tumor burden of EMD on survival of patients with MM. **(A, D)** the prognostic effects of EMD on PFS and OS of patients with MM. (**B, E)** the prognostic effects of the number of invasion sites on PFS and OS of patients with MM. (**C, F)** the prognostic effects of the tumor burden of EMD on PFS and OS of patients with MM.

Until 30 June 2022, the median follow-up was 41 months (range 2–107 m). A total of 159 patients died, and 252 patients progression during follow-up. Compared to the non-EMD and EM-B groups, the EM-E group had the worst PFS (EM-E vs. non-EMD: 14.0 vs. 31.0 m, *p* < 0.001; EM-E vs. EM-B: 14.0 vs. 26.0 m, *p* = 0.017) and OS (EM-E vs. non-EMD:26.5 vs. 68.5 m, *p* < 0.001; EM-E vs. EM-B:26.5 vs. 60.0 m, *p* < 0.001). However, there was no statistical difference in survival between the EM-B and non-EMD groups (PFS: 26.0 vs. 31.0 m, *p* = 0.258; OS:60.0 vs. 68.5 m, *p* = 0.213) (Figure 1A, D).

Patients with EMD were further divided into single-site invasion group and multi-site invasion group. The median PFS and OS of the 76 patients with single-site invasion were 27.5 and 60.0 months. Nevertheless, the median PFS and OS among the 45 patients with multi-site invasion were 24.0 and 31.0 months, respectively. Patients with multi-site invasion showed significantly shorter OS; however, no distribution difference was observed in PFS (Figure 1B, E). The cut-off Ki-67 level was defined as 15%, based on ROC curve statistics. The results showed that patients with higher Ki-67 level have shorter survival than those with lower Ki-67 expression (PFS: 48.0 vs. 19.5 m, *p* = 0.011; OS: 60.0 vs. 42.0 m, *p* = 0.045) (Figure 1C, F).

### The prognostic impact of therapy regimens and response in patients with EMDs

**Figure 2.**
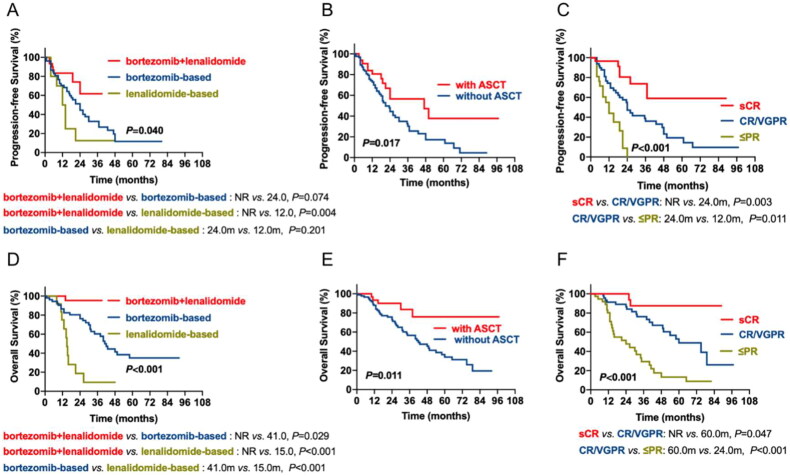
The impact of chemotherapy regimens, ASCT and curative effect on survival of patients with MM**. (A, D)** the prognostic effects of chemotherapy regimens on PFS and OS of patients with MM. (**B, E)** the prognostic effects of ASCT on PFS and OS of patients with MM. (**C, F)** the prognostic effects of the curative effect of EMD on PFS and OS of patients with MM.

Among 121 EMD patients, 53 received bortezomib-based regimens, 13 received lenalidomide-based regimens, 30 received bortezomib + lenalidomide-based regimens, and others received thalidomide-based regimens or traditional chemotherapy. The median PFS of the three subgroups was 24.0 m, 12.0 m and not reached (NR), respectively. The bortezomib + lenalidomide-based subgroup had superior PFS than the lenalidomide-based subgroup (*p* = 0.004), while no difference was detected between the bortezomib-based and lenalidomide-based subgroups. In contrast, the median OS of patients treated with bortezomib-based, lenalidomide-based, and bortezomib + lenalidomide-based regimens was 41.0 m, 15.0 m and NR. The bortezomib + lenalidomide-based subgroup had superior OS compared to the bortezomib-based (*p* = 0.029) and lenalidomide-based subgroups (*p* < 0.001), while patients who received lenalidomide-based regimens had the worst OS (lenalidomide-based vs. bortezomib-based, *p* < 0.001) (Figure 2A, D). Among all the patients with EMD, 32 underwent ASCT. The median PFS and OS of patients receiving ASCT were 47.0 m and NR, while the median PFS and OS of patients who underwent ASCT were 21.0 and 42.0 m. We found that the survival of patients with EMD was dramatically improved by ASCT (PFS: *p* = 0.017; OS: *p* = 0.011) (Figure 2B, E). We also analyzed the prognostic impact of response in patients with EMD. We observed the median PFS of sCR, CR/VGPR and ≤ PR were NR, 24.0 and 12.0 m, while the median OS was NR, 60.0 and 24.0 m, respectively. Patients who achieved sCR had the longest PFS and OS, while patients who achieved worse PFS and OS than those who achieved PR (Figure 2C, F).

### Univariate and multivariable analysis of prognostic factors of MM patients with EMDs

We further included factors including age, gender, ISS, LDH, Cr, Ca, ALB, HB, PC, CPC, 1q21 gain, del(17p), t(4;14), t(14;16), t(11;14) and extramedullary type for univariate analysis. Independent prognostic factors were assessed using multivariate analysis. We found that CPC, 1q21 gain and t(14;16) were independent prognostic factors for PFS, and the presence of LDH, CPC, del(17p), t(14;16) and extramedullary type were associated with adverse OS (Table 4).

**Table 4.** Univariate and multivariate analysis of risk factors affecting survival outcome.

Variables	PFS				OS			
	Univariate analysis		Multivariate analysis		Univariate analysis		Multivariate analysis	
	HR (95% CI)	*P* value	HR (95%CI)	*p* value	HR (95% CI)	*P* value	HR (95% CI)	*p* value
Age (≤65 vs. >65 years)	1.016 (0.721–1.432)	0.927			1,473 (0.922–2.354)	0.105		
Gender (male vs. female)	0.953 (0.689–1.317)	0.769			1.085 (0.682–1.726)	0.731		
ISS (I–II vs. III)	1.421 (1.030–1.959)	**0.032**	1.154 (0.820–1.625)	0.411	1.831 (1.151–2.914)	**0.011**	1.159 (0.656–2.048)	0.610
LDH (normal vs. elevated)	1.613 (1.030–2.524)	**0.037**	1.308 (00.813–2.104)	0.268	3.018 (1.744–5.220)	**<0.001**	1.877 (1.017–3.462)	**0.044**
Cr (≤177 vs. >177)	1.247 (0.856–1.816)	0.250			1.730 (1.050–2.849)	**0.031**	1.517 (0.807–2.852)	0.195
Ca (≤2.75 vs. >2.75)	1.556 (1.002–2.417)	**0.049**	1.301 (0.819–2.066)	0.258	2.199 (1.259–3.840)	**0.006**	1.356 (0.726–2.535)	0.339
ALB (<35 vs. ≥35)	1.047 (0.754–1.453)	0.783			1.155 (0.729–1.831)	0.540		
HB (<100 vs. ≥100)	0.846 (0.597–1.199)	0.347			0.769 (0.467–1.268)	0.303		
BMPC (≤60 vs. >60)	1.781 (1.149–2.760)	**0.010**	1.440 (0.903–2.296)	0.125	2.501 (1.435–4.359)	**0.001**	1.603 (0.868–2.962)	0.132
CPC (<0.105 vs. ≥0.105)	1.750 (1.264–2.422)	**0.001**	1.573 (1.121–2.208)	**0.009**	2.468 (1.560–3.904)	**<0.001**	2.782 (1.672–4.628)	**<0.001**
1q21 gain	1.496 (1.083–2.066)	**0.015**	1.486 (1.072–2.060)	**0.017**	1.557 (0.980–2.474)	0.061		
Del(17p)	1.745 (1.098–2.772)	**0.018**	1.539 (0.959–2.471)	0.074	2.284 (1.227–4.251)	**0.009**	1.953 (1.022–3.732)	**0.043**
t(4;14)	1.228 (0.836–1.803)	0.295			1.381 (0.811–2.349)	0.234		
t(14;16)	3.997 (1.860–8.589)	**<0.001**	4.378 (2.007–9.551)	**<0.001**	3.864 (1.551–9.626)	**0.004**	6.742 (2.571–17.682)	**<0.001**
t(11;14)	1.334 (0.792–2.245)	0.278			1.332 (0.638–2.779)	0.445		
EMD								
EM-B	1.200 (0.801–1.797)	0.376			1.014 (0.552–1.861)	0.964	1.416 (0.729–2.751)	0.305
EM-E	1.702 (0.862–3.362)	0.126			3.699 (1.749–7.822)	**0.001**	5.008 (2.116–11.855)	**<0.001**

ISS, International Staging System; LDH, lactate dehydrogenase; Cr, creatinine; Ca, corrected serum calcium; ALB, albumin; HB, hemoglobin; BMPC, bone marrow plasma cell; CPC, circulating plasma cell; ASCT, autologous stem cell transplantation. Statistically significant *p*-values are written in bold.

### Establishment of a prognostic model for MM patients with EMDs

**Figure 3.**
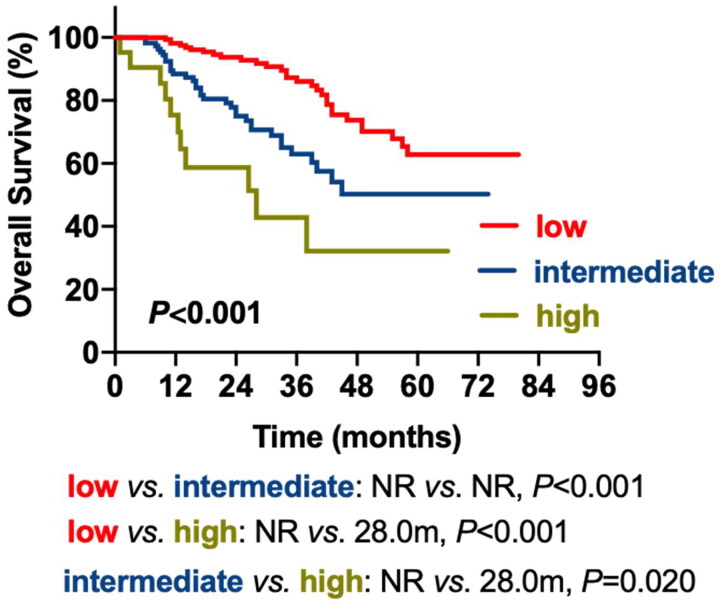
Overall survival and myeloma-specific survival according to MM-PI.

Factors including LDH, CPC, del(17p) and extramedullary type were incorporated as predictive variables in the multivariable analysis to create a prognostic model for patients with newly diagnosed MM (NDMM) (MM prognostic index; MM-PI). Since the incidence rate of t(14;16) was low in patients with MM, we excluded t(14;16) to prevent data bias. Regression analysis parameters were used to calculate the unique weighted risk scores for each independent component. Hence, weighted risk scores of 1 were assigned to LDH, del(17p) and EM-B, 2 were assigned to CPC, and 4 were assigned to EM-E. The overall risk score (MM-PI) varied from zero to eight (Table 5). We stratified the patients into three risk groups: low-risk (MM-PI:0-1), intermediate-risk (MM-PI:2-3) and high-risk (MM-PI:4-8). The median OS for the three risk groups was NR, NR and 28.0 m. The low-risk group had superior OS compared to the intermediate-risk (*p* < 0.001) and high-risk (*p* < 0.001) groups, and the high-risk group had inferior OS compared to the intermediate-risk group (*p* = 0.020) (Table 6, Figure 3).

**Table 5.** Risk scores of the independent factors for overall survival.

OS	HR (95% CI)	Assigned risk score
LDH	1.877 (1.017–3.462)	1
Del(17p)	1.953 (1.022–3.732)	1
CPC	2.782 (1.672–4.628)	2
EMD		
EM-B	1.416 (0.729–2.751)	1
EM-E	5.008 (2.116–11.855)	4

OS, overall survival; CI, confidence interval; HR, Hazard ratio; LDH, lactate dehydrogenase; CPC, circulating plasma cell.

**Table 6.** Survival data of three risk groups based on MM-PI.

	MM-PI risk score	No (%)	Median OS	Comparison	*p*
Low	0–1	183 (57.9%)	NR	vs. intermediate	**<0.001**
Intermediate	2–3	112 (35.4%)	NR	vs. high	**0.020**
High	>3	21 (6.7%)	28.0	vs. low	**<0.001**

MM-PI, multiple myeloma prognostic index; OS, overall survival; NR, not reached; bold values, mean *p*-value <0.05.

### Creation and validation of the prognostic nomogram

**Figure 4.**
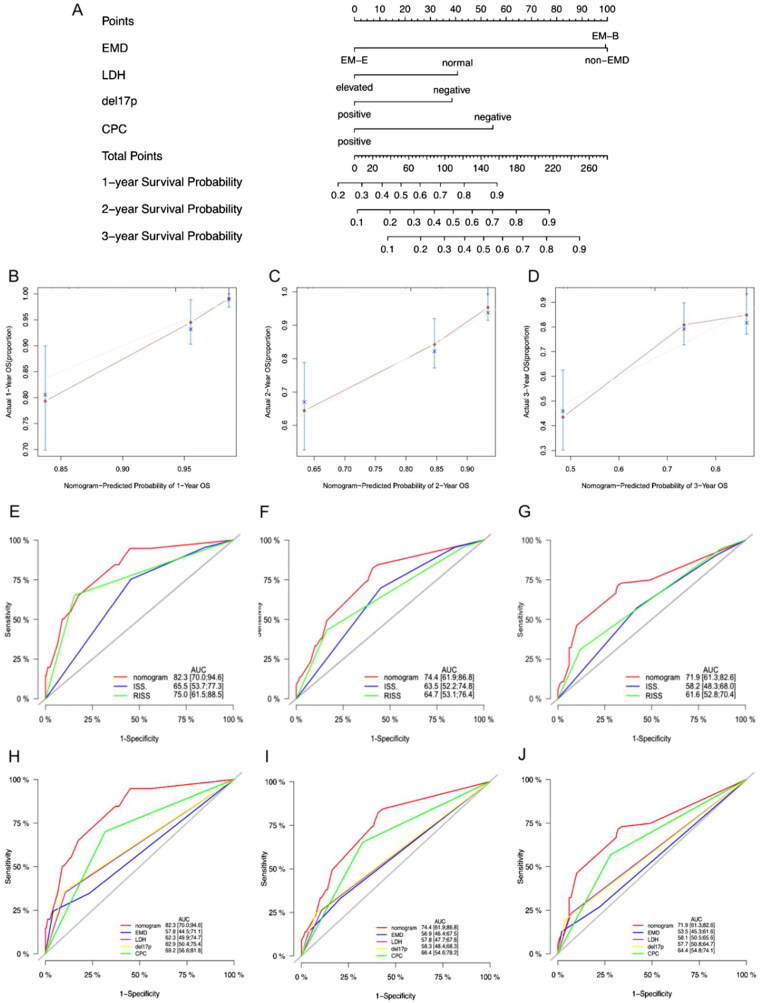
Nomogram model for 1-year, 2-year and 3-year probability of survival. (**A)** Nomogram model for patients with newly diagnosed multiple myeloma. (**B–D)** the calibration plots for the 1-year, 2-year, and 3-year OS probabilities. (**E–G)** ROC curves for ISS, RISS and the nomogram model. (**H–J)** ROC curves for EMD, LDH, del(17p), CPC and the nomogram model.

A predictive nomogram with LDH, del(17p), CPC, and extramedullary type was created based on the multivariate analysis to estimate the 1-year, 2-year and 3-year survival probability of patients with MM (Figure 4(A)). The calibration plots for the 1-year, 2-year and 3-year OS probabilities were consistent with the predicted results (Figure 4(B–D)). Receiver operating characteristic (ROC) analyses were used to assess the discrimination of the nomogram. The AUCs of the nomogram model at 1, 2, and 3 years were as followed: 0.823, 0.744 and 0.719, respectively, indicating that the prediction accuracy of the nomogram was better than that of the ISS and R-ISS (Figure 4(E–G)). The nomogram also showed better predictive efficacy than LDH, del(17p), CPC, or extramedullary type alone (Figure 4(H–J)). In summary, we established a risk model incorporating extramedullary disease, which provides an accurate and individualized survival estimation for patients with NDMM.

## Discussion

We analyzed the baseline parameters and biological features of 518 patients with NDMM in this study, especially EMD involvement. In the present study, we found that 23.3% of patients with MM had EMD invasion at the initial diagnosis, which is consistent with previous studies [2, 4, 9, 11, 13, 18, 22]. Interestingly, a lower tumor burden was found in patients with EMD than in those without EMD involvement, as indicated by a more favorable ISS stage, lower creatinine level, and lower LDH level, suggesting that EMD was attributed to severe symptoms rather than a larger disease burden. Regrettably, there was no discernible difference between EM-E and EM-B populations.

EMD has been reported as a risk factor for patients with MM in both PFS and OS [9, 11, 13, 18, 23]. Usmani et al. [13] showed a significantly shorter 5-year OS for EMD patients compared to non-EMD patients, as well as shorter 5-year PFS. In this study, we observed shorter OS and PFS in EM-E patients than in non-EMD and EM-B patients. In addition, our results showed that patients with multi-site invasion had significantly shorter OS than patients with single-site invasion, which was consistent with previous studies [24].

Fluorescence *in situ* hybridization (FISH) helps in cytogenetic abnormality detection in MM, which is of great prognostic value in MM [25]. However, the cytogenetic profile of EMD is not well understood in the published literature [12, 13, 26–29]. A few studies have shown that plasma cells harbor cytogenetic abnormalities such as del(17p), t(4;14) and t(14;16) when they progress to extramedullary disease [14]. Similarly, our data do not support the possible disruption of cytogenetic abnormalities in patients with EMD, although t(4;14) showed a slight association with EM-B occurrence. The nuclear protein Ki-67 is widely used as a biomarker of tumor proliferation and is closely related to disease progression; however, reports on the Ki-67 index in EMD are lacking [30]. In our study, a Ki-67 index > 15% was significantly correlated with inferior outcomes in patients with EMD. Furthermore, the percentage of patients with high Ki-67 index was higher in the EM-E group than in the EM-B group, which indicates that EM-E was more proliferative and invasive than EM-B.

There is no consensus regarding EMD treatment. As EMD possesses high-risk features, experts suggest that it can be treated as a high-risk MM [13, 31, 32]. Previous studies have shown that induction therapy containing bortezomib appears to be more effective in patients with EMD, whereas lenalidomide is disputable [33, 34]. The benefits of ASCT in EMD are controversial [10, 11, 35, 36]. In a study by Kumar et al. transplant-eligible MM patients with EMD achieved a lower response after ASCT, which was attributed to a higher proportion of ISS stage III and Durie Salmon stage IIIB. In addition, novel agents, such as Selinexor and anti-BCMA CAR-T cell therapy, have been reported to be effective in treating EMD [37–39]. In our study, lenalidomide-based treatment showed the worst outcomes compared to the other two groups. EMD patients who underwent ASCT showed superior PFS and OS than those who did not receive ASCT in our study.

Even in the era of novel agents, EMD involvement portends poor prognosis in MM with increased mortality and is associated with shorter PFS and OS [40–42]. In a retrospective series of 1965 MM patients, 66 patients diagnosed with EMD had a 5-year OS rate of 31%, compared to 59% for those without EMD [13]. Similarly, a lower 5-year PFS rate was found in patients with EMD (21 vs. 50%). In the study by Varettoni et al. [9] study, the presence of EMD at any time (primary or secondary) was associated with significantly shorter OS and PFS in a time-dependent analysis. However, prognostic staging systems rarely include EMD as a risk factor for MM survival prediction, even in the latest mSMART and R2-ISS models [43, 44]. In the current study, we defined EMD involvement as an independent risk factor based on the multivariate analysis. In addition to a prognostic model comprising four factors, including LDH, del(17p), CPC and extramedullary type, we also created a nomogram to predict the survival of patients with NDMM. To the best of our knowledge, our study is the first to incorporate EMD into a predictive model for MM patients and show robust OS prediction performance by AUC compared to ISS and R-ISS.

To the best of our knowledge, this study is the first predictive model incorporating EMD to predict the prognosis of NDMM. The median OS of patients with EM-E is short, which means it is crucial to investigate innovative therapeutic approaches to support patients with EMD. However, this was a retrospective single-center study with a relatively small sample size and short-term follow-up; multi-center studies based on long-term follow-up are required to validate the risk model.

## Conclusion

In conclusion, our results demonstrate the different clinical characteristics and outcomes of EM-B, EM-E and non-EMD patients. We discovered a variety of relevant prognostic variables and created a 4-factor nomogram that was reliable for predicting the course of patients with NDMM. Even in the era of novel agents, patients with EMD involvement at diagnosis still have a grave prognosis, and more research on innovative therapeutic approaches is required to mitigate the detrimental effects of EMD.

## Data Availability

The data of this study are available from the corresponding author upon request.
